# Risk of microcephaly after Zika virus infection in Brazil, 2015 to 2016

**DOI:** 10.2471/BLT.16.178608

**Published:** 2017-03-01

**Authors:** Thomas Jaenisch, Kerstin Daniela Rosenberger, Carlos Brito, Oliver Brady, Patrícia Brasil, Ernesto TA Marques

**Affiliations:** a Section of Clinical Tropical Medicine, Department of Infectious Diseases, Heidelberg University Hospital, Im Neuenheimer Feld 324, Heidelberg, 69120, Germany.; b Department of Internal Medicine, Federal University of Pernambuco, Recife, Brazil.; c Department of Infectious Disease Epidemiology, London School of Hygiene & Tropical Medicine, London, England.; d Fundacao Oswaldo Cruz, Rio de Janeiro, Brazil.; e Virology and Experimental Therapeutics Laboratory, Aggeu Magalhães Research Center, Recife, Brazil.

## Abstract

**Objective:**

To estimate the risk of microcephaly in babies born to women infected by the Zika virus during pregnancy in Brazil in an epidemic between 2015 and 2016.

**Methods:**

We obtained data on the number of notified and confirmed microcephaly cases in each Brazilian state between November 2015 and October 2016 from the health ministry. For Pernambuco State, one of the hardest hit, weekly data were available from August 2015 to October 2016 for different definitions of microcephaly. The absolute risk of microcephaly was calculated using the average number of live births reported in each state in the corresponding time period between 2012 and 2014 and assuming two infection rates: 10% and 50%. The relative risk was estimated using the reported background frequency of microcephaly in Brazil of 1.98 per 10 000 live births.

**Findings:**

The estimated absolute risk of a notified microcephaly case varied from 0.03 to 17.1% according to geographical area, the definition of microcephaly used and the infection rate. Assuming a 50% infection rate, there was an 18–127 fold higher probability of microcephaly in children born to mothers with infection during pregnancy compared with children born to mothers without infection during pregnancy in Pernambuco State. For a 10% infection rate, the probability was 88–635 folds higher.

**Conclusion:**

A large variation in the estimated risk of microcephaly was found in Brazil. Research is needed into possible effect modifiers, reliable measures of Zika virus infection and clear endpoints for congenital malformations.

## Résumé

**Objectif:**

Estimer le risque de microcéphalie chez les enfants nés de femmes infectées par le virus Zika durant la grossesse lors de l'épidémie survenue au Brésil entre 2015 et 2016.

**Méthodes:**

Nous avons obtenu du ministère de la Santé des données sur le nombre de cas de microcéphalie signalés et confirmés dans chaque État brésilien entre novembre 2015 et octobre 2016. Dans l'État de Pernambuco, l'un des plus durement touchés, des données hebdomadaires étaient disponibles d'août 2015 à octobre 2016 pour différentes définitions de la microcéphalie. Le risque absolu de microcéphalie a été calculé à partir du nombre moyen de naissances vivantes déclaré dans chaque État pendant la même durée entre 2012 et 2014 et en prenant deux taux d'infection: 10% et 50%. Le risque relatif a été estimé à partir de la fréquence habituelle de microcéphalie déclarée au Brésil, qui était de 1,98 pour 10 000 naissances vivantes.

**Résultats:**

L’estimation du risque absolu de signalement de microcéphalie allait de 0,03 à 17,1% suivant la zone géographique, la définition de la microcéphalie employée et le taux d'infection. En prenant un taux d'infection de 50%, dans l'État de Pernambuco, la probabilité de microcéphalie chez les enfants nés de mères infectées par le virus Zika pendant la grossesse était 18 à 127 fois supérieure à celle des enfants nés de mères non touchées par le virus pendant la grossesse. Avec un taux d'infection de 10%, cette probabilité était 88 à 635 fois supérieure.

**Conclusion:**

De gros écarts ont été observés au niveau de l'estimation du risque de microcéphalie au Brésil. Il est nécessaire de mener des recherches sur les éventuels facteurs modifiants, d'avoir des mesures fiables de l'infection à virus Zika et de définir clairement des critères d'évaluation des malformations congénitales.

## Resumen

**Objetivo:**

Estimar el riesgo de microcefalia en bebés nacidos de mujeres infectadas por el virus de Zika durante el embarazo en Brasil en una epidemia desatada entre 2015 y 2016.

**Métodos:**

Se obtuvieron datos del Ministerio de Salud sobre el número de casos de microcefalia notificados y confirmados en todos los estados de Brasil entre noviembre de 2015 y octubre de 2016. En el estado de Pernambuco, uno de los más afectados, se disponía de datos semanales desde agosto de 2015 hasta octubre de 2016 para diferentes definiciones de microcefalia. Se calculó el riesgo absoluto de microcefalia utilizando la cifra media de nacidos vivos registrados en todos los estados en el periodo de tiempo correspondiente entre 2012 y 2014 y asumiendo dos tasas de infección: 10% y 50%. El riesgo relativo se estimó utilizando la frecuencia de fondo de microcefalia registrada en Brasil de 1,98 por cada 10 000 nacidos vivos.

**Resultados:**

El riesgo absoluto estimado de una microcefalia notificada varió del 0,03% al 17,1% según la zona geográfica, la definición de microcefalia utilizada y la tasa de infección. Asumiendo una tasa de infección del 50%, hubo una probabilidad de microcefalia de 18 a 127 veces mayor en los niños nacidos de madres infectadas por el virus de Zika durante el embarazo, en comparación con los nacidos de madres no infectadas por el virus de Zika durante el embarazo en el estado de Pernambuco. Con una tasa de infección del 10%, la probabilidad fue de 88 a 635 veces mayor.

**Conclusión:**

En Brasil, se descubrió una gran variación en cuanto al riesgo estimado de microcefalia. Es necesario realizar una investigación sobre posibles modificadores de efectos, medidas fiables para el contagio del virus de Zika y resultados claros para malformaciones congénitas.

## ملخص

**الغرض:**

تقدير خطر وقوع الصعل (صغر الرأس) للرضع المولودين لنساء قد أُصبن بعدوى فيروس زيكا خلال فترة الحمل في البرازيل في سياق الانتشار الوبائي الذي حدث في الفترة بين عامي 2015 و2016.

**الطريقة:**

حصلنا على بيانات عن عدد حالات الإخطار بوقوع الصعل والحالات المؤكدة لوقوعه في دولة البرازيل في الفترة بين تشرين الثاني/نوفمبر 2015 وتشرين الأول/أكتوبر 2016، وذلك من خلال وزارة الصحة. وبالنظر إلى ولاية بيرنامبوكو، التي تمثل واحدة من أكثر المناطق المنكوبة بالمرض، كانت البيانات متاحةً أسبوعيًا في الفترة من أغسطس/آب 2015 حتى أكتوبر/تشرين الأول 2016 فيما يتعلق بتعريفات مختلفة للصعل. وتم احتساب الاختطار المطلق للإصابة بالصعل باستخدام متوسط عدد المواليد الأحياء الذين تم الإبلاغ بهم في كل ولاية في الفترة الزمنية المناظرة بين عامي 2012 و2014 مع افتراض معدلين للإصابة بالعدوى: 10‏% و50‏%. وتم تقدير الاختطار النسبي باستخدام المعدل السابق لتكرار الإصابة بالصعل والذي تم الإبلاغ به في البرازيل حيث بلغ 1.98 من كل 10 آلاف مولود من المواليد الأحياء.

**النتائج:**

تباين الاختطار النسبي المقدَّر فيما يتعلق بحالات الإصابة بالصعل التي تم الإخطار بها حيث تراوح بين 0.03 و‏17.1‏%، وتوقف ذلك على المنطقة الجغرافية والتعريف المستخدم للصعل ومعدل الإصابة بالعدوى. وبافتراض وصول معدل الإصابة إلى ‏50%، ارتفع احتمال الإصابة بالصعل لدى الأطفال المولودين لأمهات مصابات بعدوى فيروس زيكا بمقدار 18 – 127 مرة أثناء فترة الحمل في بيرنامبوكو. أما بالنظر إلى معدل الإصابة بالعدوى البالغ 10‏%، فقد ارتفع هذا الاحتمال بمقدار 88 – 635 مرة.

**الاستنتاج:**

تم التوصل إلى وجود تباين كبير في الخطر الذي تم تقديره فيما يتعلق بالإصابة بالصعل في البرازيل. ويحتاج الأمر إلى إجراء البحث العلمي بشأن العوامل المحتملة لتغيير التأثير، وما يمكن الاعتماد عليه من الإجراءات الواجب اتخاذها حيال عدوى فيروس زيكا ونقاط نهاية واضحة للتشوهات الولادية.

## 摘要

**目的:**

旨在估测 2015 至 2016 年流行病蔓延期间孕期感染寨卡病毒女性所生婴儿的小头症患病风险。

**方法:**

我们从卫生部获取了 2015 年 11 月至 2016 年 10 月期间巴西各州通报及确认的小头症病例的数据。 从伯南布哥州,患病重灾区之一,可获取其 2015 年 8 月至 2016 年 10 月期间具有不同定义的小头症的周数据。 小头症绝对患病风险的计算采用各州在 2012 年至 2014 年相应时间段内呈报的活产婴儿的平均数量,并假设两个感染率为: 10% 和 50%。 相对风险采用巴西每 10,000 个活产婴儿中 1.98 例小头症患儿这一报道背景频率进行估测。

**结果:**

根据地理区域、所采用的小头症定义以及感染率的不同,所呈报小头症的估测绝对患病风险也有所不同,其变化范围为 0.03 至 17.1%。 在伯南布哥州,假设感染率为 50%,相较于未在孕期感染寨卡病毒的母亲所生的婴儿,孕期感染寨卡病毒母亲所生婴儿的小头症患病可能性高出前者 18-127 倍。 若感染率为 10%,该可能性则高出前者 88-635 倍。

**结论:**

经发现,在巴西,估测的小头症患病风险有较大差异。 仍需对寨卡病毒感染及先天性畸形确切终结点的可能影响效应及可靠措施进行研究。

## Резюме

**Цель:**

Оценить риск микроцефалии у младенцев, рожденных от женщин, которые заразились вирусом Зика во время беременности в Бразилии в период эпидемии между 2015 и 2016 годами.

**Методы:**

Министерство здравоохранения предоставило данные о количестве зарегистрированных и подтвержденных случаев микроцефалии в каждом штате Бразилии в период с ноября 2015 года по октябрь 2016 года. По штату Пернамбуку, который был одним из наиболее пострадавших штатов, были доступны еженедельные данные для различных определений микроцефалии в период с августа 2015 года по октябрь 2016 года. Абсолютный риск микроцефалии рассчитали с помощью среднего количества живорожденных младенцев, зарегистрированных в каждом штате в соответствующий период времени между 2012 и 2014 годами, принимая во внимание два уровня заболеваемости: 10 и 50%. Для оценки относительного риска использовалась зарегистрированная фоновая частота микроцефалии в Бразилии, составлявшая 1,98 на 10 000 живорожденных младенцев.

**Результаты:**

Полученный абсолютный риск зарегистрированного случая микроцефалии колебался от 0,03 до 17,1%, в зависимости от географического района, используемого определения микроцефалии и уровня заболеваемости. При предположительном уровне заболеваемости, равном 50%, вероятность развития микроцефалии у детей, рожденных от матерей с вирусной инфекцией Зика во время беременности, по сравнению с детьми, рожденными от матерей без вирусной инфекции Зика во время беременности, была в 18–127 раз выше в штате Пернамбуку. Для 10-процентного уровня заболеваемости вероятность была в 88–635 раз выше.

**Вывод:**

В Бразилии был обнаружен большой разброс в оценке риска развития микроцефалии. Необходимы дальнейшие исследования возможных модификаторов эффекта, надежных показателей вирусной инфекции Зика и четких конечных критериев оценки для врожденных пороков развития.

## Background

The Zika virus was initially identified in rhesus monkeys from the Zika forest in Uganda in 1947.^1^ However, it was only in 2007 that it was first reported outside Africa and Asia,^2^ when an epidemic occurred on Yap Island, in the Federated States of Micronesia.^3^ During 2013 and 2014, there was another epidemic in French Polynesia.^4^ With the virus’s emergence in Brazil in 2015, a new era began. In October 2015, an increase in the number of babies born with microcephaly (referred to as microcephaly cases) was noticed in Recife in north-east Brazil; numbers continued to increase throughout the following months and reached an unprecedented total of 1912 notified cases with microcephaly by 30 April 2016.^5^ In the absence of an alternative explanation and because of the temporal clustering observed, it was hypothesized that there was a causal association with Zika virus infection during pregnancy.^6^^,^^7^ As the evidence accumulated, the Brazilian Government suspected this association early onand declared a national public health emergency on 11 November 2015.^8^ Interestingly, after reports of the possible link between Zika virus infection and microcephaly had appeared in north-east Brazil, researchers in French Polynesia reanalysed their data and also observed this association.^9^^,^^10^ In addition, the possible association was highlighted in November and December 2015 by the Centers for Disease Control and Prevention in the United States of America, the European Centre for Disease Prevention and Control and the World Health Organization (WHO).^7^^,^^11^^,^^12^ On 1 February 2016, WHO declared that the clusters of microcephaly and other neurological disorders in babies constituted a Public Health Emergency of International Concern.^13^

The Zika virus’s potential for perinatal transmission had already been documented in 2014.^14^ By 2016, the Zika virus had been identified in the amniotic fluid of fetuses with microcephaly in Brazil^15^ and isolated cases of congenital malformations associated with the virus started to appear in other parts of the world, such as Slovenia^16^ and Hawaii,^17^^,^^18^ among individuals who had travelled to Brazil during early pregnancy. To our knowledge, never before in the history of public health have countries advised their populations to postpone planned pregnancies, as occurred, for example, in Brazil, Colombia, El Salvador and Jamaica.^19^^–^^22^ During the first half of 2016, the accumulating evidence was considered strong enough to support an etiological link between Zika virus infection and birth defects.^23^

Still, the exact risk of microcephaly and other congenital malformations linked to Zika virus infection during pregnancy remains unknown. The aim of this study was to estimate the risk of microcephaly – the most severe congenital malformation associated with Zika virus infection – in babies born to women in Brazil who were infected during pregnancy by examining the number of live births and the number of microcephaly cases reported in different states across the country. In addition, for Pernambuco State in north-east Brazil, where weekly figures on microcephaly cases were available for different definitions of the condition, we investigated absolute and relative risks in more detail.

## Methods

For notification purposes, microcephaly was defined by the Brazilian Ministry of Health (after 8 December 2015) as a head circumference in full-term babies less than 32 cm and, in preterm babies, more than 2 standard deviations below the mean as indicated by the Fenton scale.^24^ The sensitivity and specificity of this definition for identifying confirmed microcephaly, as determined by imaging, were reported to be 86.0% and 93.8%, respectively, based on a series of 31 microcephaly cases from 10 states in Brazil.^24^ Subsequently, the cases notified using the Brazilian Ministry of Health definition were reclassified using more specific definitions, such as the WHO InterGrowth standards.^25^ For our study, we obtained data on microcephaly cases reported in each state in Brazil from the Ministry of Health and determined the risk of a notified or confirmed case of microcephaly in different geographical regions between 8 November 2015 and 15 October 2016,^26^ except in Pernambuco State, where the study period was from 1 August 2015 to 15 October 2016. In particular, we compared risks in the north and south of the country. However, not all notified cases had been referred for confirmation by the time of data analysis: the proportion referred for confirmation ranged from 10% in Pará State to 96% in Piauí State (median: 76%). Consequently, we also considered the number of predicted confirmed cases, which was derived by multiplying the number of notified cases by the proportion of notified cases referred for confirmation that had been confirmed.

In estimating the risk of microcephaly in a particular state during a specific time period, we used as the denominator the average of the number of live births that occurred in the same period in that state in the three preceding years: 2012, 2013 and 2014.^27^ We assumed that the proportion of women infected by the Zika virus during pregnancy lay between 10 and 50%, as suggested by estimates from recent epidemics in the Pacific Islands, and we used the upper and lower bounds of this range to estimate risks. To calculate relative risks, we used the background frequency of microcephaly in Brazil, which was reported to be 1.98 (95% confidence interval, CI: 1.48–2.27) per 10 000 live births between 1982 and 2013.^28^ Confidence limits for relative risks were derived by dividing the lower confidence bound of the estimated absolute risk by the upper confidence bound of the observed background frequency and by dividing the upper confidence bound of the estimated risk by the lower bound of the background frequency, respectively. In Pernambuco State, where the expected number of live births during the study period was 171 402, the number of microcephaly cases reported was 5 in 2011, 9 in 2012, 10 in 2013 and 12 in 2014,^6^ which corresponds to a considerably lower frequency than the background frequency we used to estimate relative risk.

## Results

**Fig. 1:**
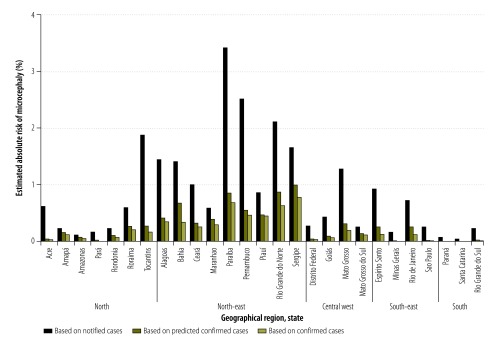
Estimated absolute risk of microcephaly in a baby born to a woman infected by the Zika virus during pregnancy, assuming a 50% infection rate, by state, Brazil, 8 November 2015 to 15 October 2016

Note: The number of predicted confirmed cases was derived by multiplying the number of notified cases by the proportion of notified cases referred for confirmation that had been confirmed.

**Fig. 2:**
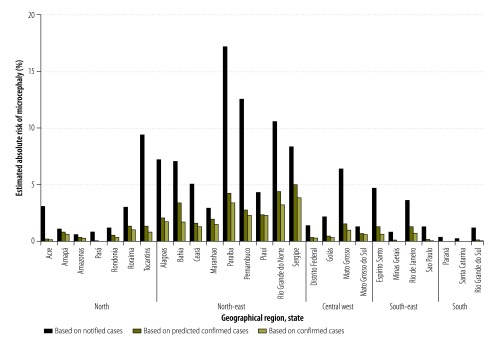
Estimated absolute risk of microcephaly in a baby born to a woman infected by the Zika virus during pregnancy, assuming a 10% infection rate, by state, Brazil, 8 November 2015 to 15 October 2016

Note: The number of predicted confirmed cases was derived by multiplying the number of notified cases by the proportion of notified cases referred for confirmation that had been confirmed.

By assuming the proportion of women infected by the Zika virus during pregnancy was 50%, our estimate of the absolute risk of a notified microcephaly case in a baby born to a woman infected during pregnancy between 8 November 2015 and 15 October 2016 ranged from 0.03% in Santa Catarina State in southern Brazil to 3.42% in Paraíba State in north-east Brazil; the risk was 0.72% in Rio de Janeiro State and 2.51% in Pernambuco State (Fig. 1). When the proportion of women infected was assumed to be 10%, the corresponding estimated risks were substantially higher: 0.16% in Santa Catarina State, 17.11% in Paraíba State, 3.61% in Rio de Janeiro State and 12.57% in Pernambuco State (Fig. 2). For all estimates, we used the Brazilian Ministry of Health’s definition of a notified microcephaly case. In addition, the estimated absolute risk of a predicted confirmed microcephaly case in a baby born to a woman infected during pregnancy, assuming a 50% infection rate, ranged from 0.006% in Paraná State in southern Brazil to 0.99% in Sergipe State in north-east Brazil; the risk was 0.25% in Rio de Janeiro State and 0.54% in Pernambuco State (Fig. 1). Assuming a 10% infection rate, the corresponding estimated risks were 0.03%, 4.96%, 1.25% and 2.72% in the four states, respectively (Fig. 2). Table 1 lists the estimated absolute risks of notified, predicted confirmed and confirmed cases during the study period in six states: Rio de Janeiro, Pernambuco, Paraiba, Santa Catarina, Sergipe and Paraná.

**Table 1:** Estimated absolute risk of microcephaly in a baby born to a woman infected by the Zika virus during pregnancy, Brazil, 8 November 2015 to 15 October 2016

State	No. of estimated live births^a^	Estimated absolute risk of microcephaly,^b^ (%)							
		Assuming a 10% Zika virus infection rate during pregnancy				Assuming a 50% Zika virus infection rate during pregnancy		
		Notified cases	Predicted confirmed cases^c^	Confirmed cases		Notified cases	Predicted confirmed cases^c^	Confirmed cases
Rio de Janeiro	213 745	3.61	1.25	0.63		0.72	0.25	0.13
Pernambuco^d^	171 402	12.57	2.72	2.28		2.51	0.54	0.46
Paraíba	53 586	17.11	4.25	3.40		3.42	0.85	0.68
Santa Catarina	85 452	0.16	0.05	0.05		0.03	0.01	0.009
Sergipe	32 225	8.29	4.96	3.85		1.66	0.99	0.77
Paraná	147 382	0.33	0.03	0.03		0.07	0.006	0.005

^a^ The number of live births was estimated by averaging the number reported over the same time period in the state in 2012, 2013 and 2014.

^b^ Microcephaly as defined by the Brazilian Ministry of Health.

^c^ The number of predicted confirmed cases was derived by multiplying the number of notified cases by the proportion of notified cases referred for confirmation that had been confirmed.

^d^ The study time period for Pernambuco was 1 August 2015 to 15 October 2016.

**Fig. 3:**
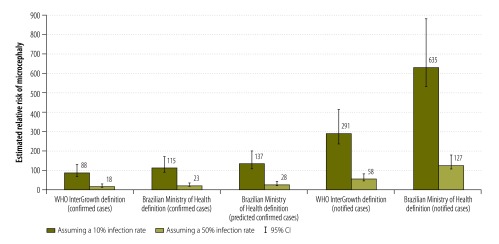
Relative risk of microcephaly in a baby born to a woman infected by the Zika virus during pregnancy, by microcephaly definition and infection rate, Pernambuco State, Brazil, 1 August 2015 to 15 October 2016

In Pernambuco State, 2155 microcephaly cases were notified between 1 August 2015 and 15 October 2016, 988 of which satisfied WHO InterGrowth standards for microcephaly.^25^ Of the 988 babies, 375 had a head circumference more than 3 standard deviations below the relevant mean and 613 had a circumference between 2 and 3 standard deviations below the mean. Details of whether cases in Pernambuco State met InterGrowth standards were available for each week throughout the course of the epidemic.^5^ In the three peak months of the epidemic from October to December 2015 (i.e. in epidemiological weeks 40 to 52, from 4 October 2015 to 2 January 2016), 448 cases were documented; for comparison, 328 cases were documented over 26 weeks before and after the peak (i.e. in epidemiological weeks 31 to 39 in 2015, from 2 August 2015 to 3 October 2015, and in weeks 1 to 17 in 2016, from 3 January 2016 to 30 April 2016). Table 2 lists the estimated absolute risk of notified, predicted confirmed and confirmed cases for different definitions of microcephaly and for infection rates of 10% and 50%. Depending on the definition of microcephaly used, the estimated relative risk of microcephaly in Pernambuco State varied between 18 and 127 assuming a 50% infection rate and between 88 and 635 assuming a 10% infection rate (Fig. 3). This is equivalent to an 18–127 fold (88–635 fold) higher probability of microcephaly in children born to mothers with ZIKV infection during pregnancy compared with children born to mothers without ZIKV infection during pregnancy.

**Table 2:** Estimated absolute risk of microcephaly in a baby born to a woman infected by the Zika virus during pregnancy, Pernambuco State, Brazil, 1 August 2015 to 15 October 2016

Definition of microcephaly	Estimated absolute risk of microcephaly, (%)						
	Assuming a 10% Zika virus infection rate during pregnancy				Assuming a 50% Zika virus infection rate during pregnancy		
	Notified cases	Predicted confirmed cases^a^	Confirmed cases		Notified cases	Predicted confirmed cases^a^	Confirmed cases
Brazilian Ministry of Health definition	12.57	2.72	2.28		2.51	0.54	0.46
WHO InterGrowth definition	5.76	ND	1.74		1.15	ND	0.35

ND: not determined; WHO: World Health Organization.

^a^ The number of predicted confirmed cases was derived by multiplying the number of notified cases by the proportion of notified cases referred for confirmation that had been confirmed.

CI: confidence interval.

Note: The number of predicted confirmed cases was derived by multiplying the number of notified cases by the proportion of notified cases referred for confirmation that had been confirmed.

## Discussion

We found the estimated risk that a baby born to a woman infected by the Zika virus during pregnancy would have microcephaly varied substantially across Brazil. In particular, the risk was affected by: (i) geographical area; (ii)the definition of microcephaly used; and (iii) the percentage of women assumed to have been infected by the virus during pregnancy. In the epidemics in Yap Island in 2007 and in French Polynesia during 2013 and 2014, the seroprevalence, which reflected the proportion of the population exposed to the Zika virus, was 73% and 50 to 66%, respectively, for outbreaks that lasted 4 and 14 months, respectively.^3^^,^^29^ Corresponding data are not yet available for the 2015 Zika epidemic in Brazil. The high seroprevalence reported after the limited-duration epidemics in the Federated States of Micronesia and French Polynesia suggest that the virus is easily readily transmitted. Consequently, herd immunity could have built up quickly and blocked further transmission. In both countries, serological tests were performed after the epidemics to determine the seroprevalence of antibodies to the Zika virus as well as to related flaviviruses, including dengue viruses. Although the possible presence of cross-reacting antibodies was taken into account when interpreting the results,^3^^,^^29^ cross-reactivity may still have led to an overestimate of the seroprevalence of Zika virus antibodies in these two countries. Consequently, the reported seroprevalence in the Federated States of Micronesia and French Polynesia may be higher than would be expected in the southern states of Brazil, where there is a substantial seasonal variation in viral transmission. On the other hand, the possibility that an epidemic will undergo a stochastic die-out is greater in isolated, small, island populations;^30^ the true seroprevalence may, therefore, have been underestimated, assuming the infection reached an equilibrium.

We observed a large variation in the risk of microcephaly between federal states in Brazil: the highest risks occurred in the north-east, whereas lower figures were observed inland and in the south. Similar to many states in Brazil with a moderate risk, the absolute risk of microcephaly linked to Zika virus infection during the first trimester in French Polynesia was also estimated to be around 1% in a retrospective analysis.^9^ Some Brazilian states might not yet have reported microcephaly cases because the epidemic occurred late in 2015 or because they only had imported cases. However, the epidemics in Pernambuco and Rio de Janeiro States peaked almost at the same time in the spring of 2015.^25^^,^^31^ The observation that the estimated risk was substantially higher in north-east Brazil than in Rio de Janeiro State, therefore, merits further attention. The possibility that cofactors or effect modifiers can play a role should be investigated in future studies.

The definitions of microcephaly used in Brazil have changed and more specific criteria based on WHO InterGrowth standards have been adopted. However, the sensitivity of these more specific criteria may be lower.^24^ Current estimates of the sensitivity and specificity of different definitions of microcephaly are based on relatively small samples^24^ and need to be validated in larger studies. In our study, we focused on the risk of microcephaly. However, the Zika virus may be associated with a wider range of congenital abnormalities and adverse pregnancy outcomes, including placental diseases that depend on gestational age at the time of infection. An interim analysis from Rio de Janeiro found that 29% of 42 women who had a confirmed Zika virus infection during pregnancy had babies with congenital abnormalities that were detected by ultrasound, including one baby with microcephaly.^32^

Our study was limited by the fact that the number of microcephaly cases was updated frequently and because definitions of microcephaly changed over time, both of which could have resulted in a substantial variation in the estimated risk of microcephaly. Furthermore, we had to make assumptions about the proportion of women infected by the Zika virus during pregnancy, which had a large influence on the estimated risk. Community-based seroprevalence studies of women of child-bearing age are needed to gain a better understanding of the proportion of the population infected over the course of a Zika virus epidemic.

In the absence of robust estimates of absolute and relative risks of microcephaly, cohort studies are urgently needed to determine the risk in pregnant women at different gestational ages. Moreover, microcephaly should not be the only measurement. Future studies should also evaluate the influence of potential cofactors and effect modifiers, given the wide geographical variation in risk we observed. Nevertheless, preliminary estimates of the magnitude and range of absolute and relative risks, such as those reported here, are valuable for designing future cohort studies.
